# Results from Two HPV-Based Cervical Cancer Screening-Family Planning Integration Models in Malawi: A Cluster Randomized Trial

**DOI:** 10.3390/cancers15102797

**Published:** 2023-05-17

**Authors:** Jennifer H. Tang, Fan Lee, Maganizo B. Chagomerana, Kachengwa Ghambi, Patani Mhango, Lizzie Msowoya, Tawonga Mkochi, Irene Magongwa, Eneli Mhango, Jacqueline Mbendera, Eunice Mwandira, Erik Schouten, Leah Gardner, Jennifer S. Smith, Luis Gadama, Lameck Chinula

**Affiliations:** 1 Department of Obstetrics & Gynecology, University of North Carolina at Chapel Hill, 4002 Old Clinic Building, Chapel Hill, NC 27599, USA; 2 UNC Project-Malawi, Tidziwe Center, Kamuzu Central Hospital, 100 Mzimba Road, Lilongwe 207233, Malawi; 3 Department of Medicine, University of North Carolina at Chapel Hill, 321 South Columbia Street, Chapel Hill, NC 27599, USA; 4 Department of Obstetrics & Gynaecology, Kamuzu University of Health Sciences, Mahatma Gandhi Road, Blantyre 312225, Malawi; 5 Department of Epidemiology, University of North Carolina at Chapel Hill, McGavran-Greenberg Hall, Chapel Hill, NC 27599, USA

**Keywords:** HPV self-collection, cervical cancer, family planning

## Abstract

**Simple Summary:**

Our study evaluated which of two HPV-based cervical cancer screening–family planning integration models would result in a higher proportion of eligible women ever being screened for cervical cancer in our targeted communities in Malawi. We found that the model that offered HPV self-collection in both the local health clinic and through community health workers resulted in a significantly higher proportion of eligible women ever being screened in that model’s targeted communities, when compared to the model that only offered HPV self-collection at the local health clinic. In addition, women in the clinic–community integration model were more likely to be using modern family planning services. Therefore, we recommend that countries and programs aiming to increase the proportion of eligible women ever screened for cervical cancer integrate HPV self-collection with family planning services in both clinic- and community-based settings to reach women that have never been screened for cervical cancer.

**Abstract:**

We conducted a cluster randomized trial of two models for integrating HPV self-collection into family-planning (FP) services at 16 health facilities in Malawi between March 2020–December 2021. Model 1 involved providing only clinic-based HPV self-collection, whereas Model 2 included both clinic-based and community-based HPV self-collection. An endline household survey was performed in sampled villages and households between October-December 2021 in the catchment areas of the health facilities. We analyzed 7664 surveys from 400 villages. Participants from Model 2 areas were more likely to have ever undergone cervical cancer screening (CCS) than participants from Model 1 areas, after adjusting for district, facility location (urban versus rural), and facility size (hospital versus health center) (adjusted odds ratio = 1.73; 95% CI: 1.29, 2.33). Among participants who had ever undergone CCS, participants from Model 2 were more likely to report having undergone HPV self-collection than participants from Model 1 (50.5% versus 22.8%, *p* = 0.023). Participants from Model 2 were more likely to be using modern FP (adjusted odds ratio = 1.01; 95% CI: 1.41, 1.98) than Model 1 participants. The integration of FP and HPV self-collection in both the clinic and community increases CCS and modern FP uptake more than integration at the clinic-level alone.

## 1. Introduction

Cervical cancer is the fourth most common cancer among women worldwide, accounting for over 600,000 new cases and over 340,000 new deaths in 2020 [1]. However, in much of Sub-Saharan Africa (SSA), which has high rates of HIV, it is the most common cancer among women [1]. Malawi, a country in SSA with a HIV infection rate of 9.4% among women aged 15–49 years, has the world’s highest cervical cancer incidence and mortality rates [1,2].

Almost all cervical cancer is the result of an infection with high-risk human papillomavirus (hrHPV) [3]. Therefore, primary prevention for cervical cancer can be achieved on a national level through national HPV vaccination, particularly among girls aged 9–14 years, before they are exposed to HPV through sexual activity [4]. However, many countries only recently began rolling out HPV vaccination and as of June 2020, only 107 (55%) of the 194 World Health Organization (WHO) Member States were considered to have introduced HPV vaccination in their country [5]. Therefore, secondary prevention of cervical cancer through screening with a high-performance test, followed by treatment for those who are screen-positive, is also critical to prevent cervical cancer [6].

The WHO currently recommends using HPV detection as the primary screening test, rather than visual inspection with acetic acid (VIA) or cytology, starting at age 30 years for the general population of women and at age 25 years for women living with HIV (WLWH) [6]. In some programs, women who screen HPV-positive undergo immediate treatment, known as the “HPV screen-and-treat” approach, where the decision to treat is based on the HPV test only. In contrast, other programs use the “HPV screen, triage and treat” approach, where the decision to treat an HPV-positive woman is based on the result of a positive second “triage” test, usually VIA [6]. With either approach, ablative therapy can be performed on the same day among eligible women if the HPV test results can be given to the patient on the same day, minimizing loss-to-follow-up after screening. The GeneXpert^®^ HPV test (Cepheid Inc, Sunnyvale, CA, USA) is one such test that allows HPV results to be given on the same day because samples are non-batched and each take only 1–2 h to complete [7].

HPV self-collection is an HPV-based screening strategy that is increasingly used to improve access to cervical cancer screening in at least 27 countries around the world, including both high-income and low-and-middle-income countries (LMIC) across six continents [8]. This strategy is endorsed by the WHO, which notes that “HPV self-collection should be made available as an additional approach to sampling in cervical cancer screening services for individuals aged 30–60 years” [9]. HPV self-collection is highly acceptable among women, regardless of age, income, or country of residence [10], and is similarly accurate as provider-collected samples when highly sensitive hrHPV assays are used [11].

Therefore, we designed a cluster randomized feasibility trial of two different implementation models for integrating HPV self-collection into family planning (FP) services in Malawi. Model 1 involved only clinic-based HPV self-collection, whereas Model 2 included both clinic-based and community-based HPV self-collection. Our primary hypothesis was that the Model 2 facilities would have a higher proportion of eligible women undergo cervical cancer screening (CCS) in their catchment areas during our study than in the catchment areas of the Model 1 facilities [12]. A secondary hypothesis was that the Model 2 facilities would have a higher proportion of eligible women who received FP services in their catchment areas during the study than in the catchment areas of the Model 1 facilities.

## 2. Materials and Methods

### 2.1. Study Design and Population

This study was a hybrid type 2 cluster randomized feasibility trial [12], with 16 health facilities from two districts in Malawi (Lilongwe and Zomba Districts) assigned to either Model 1 or Model 2 (Table 1). In June 2019, we began working with the Malawi Ministry of Health (MoH) to modify existing MoH registers and documents for facility cervical cancer screening and preventive therapy (CCSPT), facility FP, and community FP services. We also worked with the Lilongwe and Zomba District Health Management Teams to determine which eight facilities in each of their districts should be included in the study, based on their needs and practical constraints, such as if it had a lab, and if so, if it had sufficient space to store and operate a GeneXpert machine. To evaluate implementation of our models at multiple types of facilities, each district was asked to identify one central or district hospital, one Christian Health Association of Malawi (CHAM) hospital, two Urban Health Centers, and four rural health facilities for inclusion in the study.

In November and December 2019, we conducted educational talks in the catchment areas of the 16 health facilities and held stakeholder-engagement meetings with community leaders and heath-facility staff so that they were aware of our study activities. During this time period, we purchased GeneXpert^®^ machines for each of the facilities and installed solar panels and back-up uninterruptable power supply (UPS) systems for each machine since power outages in Malawian facilities are common. We provided training for lab technicians from our study facilities on how to perform HPV testing on the GeneXpert^®^ machines, as well as on our study protocol and related documents. We also provided training for providers (medical officers, clinical officers, and nurses) from our study facilities in the Malawi MoH’s one-week VIA and thermal ablation course, followed by a one-day training on our HPV self-collection algorithm and procedures.

For facilities randomized to Model 2, we also trained their health surveillance assistants (HSAs) on how to offer HPV self-collection in the communities. HSAs are a cadre of Malawi’s government-employed community-health workers who are assigned to provide primary health services to specific villages that are located 5 km or more from the health facility at which they are based [13]. HSAs typically provide services to a population of ~1200 people by visiting these communities at least once a month through outreach clinics (typically held in a local school or other public buildings with nurses from the facilities), village clinics (typically held in the HSA’s home without nurses), and occasionally, home visits. In addition to other health services, HSAs can provide the depot medroxyprogesterone acetate (DMPA) injectable, oral contraceptives, and condoms in the community if they undergo the Malawi MoH’s five-day FP training for HSAs. Since one of our project’s objectives was to improve FP access, we also provided this FP training to Model 2 HSAs who had never completed it.

Our HPV self-collection strategy was integrated into FP services at the facility-level by providing educational talks to women in the FP clinic waiting room about cervical cancer, the importance of screening for it, and the option to undergo HPV self-collection at the clinic while they waited to access FP and other services. Women who were eligible for cervical cancer screening were then offered HPV self-collection kits, which included a Viba-Brush^®^ (Rovers Medical Devices, Amsterdam, The Netherlands) and an empty tube labeled with their name, enclosed within a plastic zipper bag. Eligibility criteria for cervical cancer screening were: (1) age 25–49 years; (2) no prior total hysterectomy, (3) no history of cervical cancer, and (4) not pregnant.

Women interested in undergoing HPV self-collection were given instructions and a pictorial guide (please see File S1) on how to use the self-collection kit, which included 10 steps: (1) making sure that they had the brush and a small tube labeled with their name; (2) finding a private place to collect the sample and washing their hands with soap and water; (3) removing the brush from its envelope and trying not to touch the white brush tip with their hands; (4) standing, sitting, lying down, or squatting with their legs apart; (5) holding the lips of the vagina open with one hand and placing the brush into the vagina with the other hand until slight pressure was felt; (6) gently turning the brush around five times while in the vagina and then slowly pulling out the brush from the vagina; (7) holding the brush and removing the cap from the labeled tube; (8) pushing the clear plastic on the handle down so that the white brush tip falls into the tube and throwing away the blue handle; (9) capping the tube with the brush inside, screwing its lid on tightly, washing their hands again, and placing the tube in the bio-hazard bag provided; and (10) returning the plastic bag with the tube/brush to the provider.

Once a woman completed HPV self-collection, she was encouraged to wait to receive her HPV test results if possible, so that she could undergo treatment on the same day if her HPV test was positive. The tubes were transported to the lab for the woman’s facility, where HPV testing was performed on them as per GeneXpert^®^ instructions, on the same day whenever possible. We used the HPV-and-treat strategy [6], i.e., if a women tested HPV-positive, she was offered VIA, and if she did not have cervical lesions or lesions ineligible for ablative therapy, she would undergo thermal ablation immediately after VIA if she consented to it. Women who underwent thermal ablation were advised to return for follow-up cervical cancer screening in one year. Eligibility criteria for thermal ablation treatment were: (1) no cervical lesions or lesions that can be seen in their entirety and do not cover >75% of the cervix; (2) no lesions that extended into the endocervix or to the vaginal wall; (3) visible squamocolumnar junction; (4) not currently pregnant or <3 months from delivery; (5) no concern for cervical cancer on exam; (6) no polyps or other cervical lesions that would prevent the probe from contacting the cervix; (7) not currently menstruating; and (8) no concern for cervicitis.

For facilities randomized to Model 2, we also integrated HPV self-collection into the HSAs’ community-based clinics, so that they would offer both FP services and HPV self-collection to eligible women in the community. The HSAs would bring HPV self-collection kits into the community and then return the collected tubes/brushes within seven days of self-collection to their facility, where HPV testing would be performed on the samples. Women who underwent HPV self-collection in the community were given the option to go to their local health facility to get their HPV results or have the HSA bring the result to their next monthly visit to their village. Women who received an HPV-positive result were referred to the facility for VIA and treatment upon indication. For further details about other study components that will be analyzed in separate manuscripts, please refer to the study protocol paper [12].

### 2.2. Study Outcomes and Variables

Our primary and secondary outcomes were originally as follows: (a) proportion of eligible women aged 25–50 years who received CCS during project implementation (Primary Outcome); (b) proportion of treatment-eligible women who received thermocoagulation during project implementation (Secondary Outcome #1); and (c) proportion of women aged 15–50 years using a modern FP method (Secondary Outcome #2).

However, due to the difficulty that participants had with remembering the approximate date of their last CCS, we were not able to calculate the proportions of women who received CCS during study implementation for our primary outcome. Since women were able to recall if they had ever been screened for CCS, we used this response as our primary outcome instead.

The study outcomes were evaluated through an endline household survey (EHS), which was performed between October to December 2021, approximately 12 months after all facilities had begun implementing our study. The EHS was administered to women living in the catchment areas of all 16 facilities except for Zomba Central Hospital because its catchment area encompassed other districts in southern Malawi not included in our study. Inclusion criteria for the EHS were: (1) women between the ages of 15–50 years, (2) no history of total hysterectomy prior the initial study implementation in March 2020, and (3) ability to give informed consent in English or Chichewa (the local language).

The EHS included the following study variables: basic demographics, reproductive health history, HIV status, distance to the nearest health facility, prior CCS (Primary Outcome), CCS since March 2020, ever use of HPV self-collection, ever receipt of thermal ablation (Secondary Outcome #1), prior FP use, FP method use since March 2020, and current FP method use (Secondary Outcome #2). In Malawi, all medical information is recorded in an individual’s health passport. If a participant had her health passport available and was willing to allow the study research assistant to review it, her FP-method use and prior CCSPT responses were verified with the health passport.

### 2.3. Study Sampling for the Endline Household Survey

The target sample size was 8000 women from 400 villages selected proportionally from the 16 facility catchment areas. To develop our sampling strategy for the EHS, village data and enumeration maps for the 16 facility catchment areas were gathered from Malawi National Statistics Office, the Lilongwe and Zomba District Health Offices, and the HSAs from each facility. A two-stage sampling plan was then followed. For Stage 1, our biostatistician (MC) selected a random sample of 400 villages to be interviewed from each facility’s catchment area, which was proportional to the size of the catchment area of each facility. Household lists for each randomly sampled village were then obtained from their HSAs and village leaders. If a village could not be interviewed (because it was found to not actually be in the catchment area of the facility, it no longer existed and/or had merged with another village, it was inaccessible because it required a boat to reach, or because its leaders did not allow us to interview there), a village was replaced with another similar village nearby.

Then, for Stage 2, our EHS research assistants (RAs) randomly selected 20 households per village to interview. Specifically, on each day of data collection, teams of two RAs arrived at their assigned village and verified the household list with the village HSA. A sampling fraction (*h*) was calculated by dividing the total number of households in the village by the number of households to survey (n = 20). The first household was identified by selecting a number between 1 and *h*. Random number selection was done in the field by writing numbers on pieces of paper, folding them up, placing them in a container and mixing before drawing one out at random. The second household to sample was determined by the initial number + h and sampling was proceeded in this manner with every *h*th household being sampled. When individual(s) in selected households were unavailable or not eligible to participate, that household is replaced with the next household in the direction of travel. HSAs were compensated for their field assistance at the equivalent of about USD$5.

At each selected household, all potential participants were counseled about the purpose of the study and its procedures. Those interested were screened for eligibility and if eligible, two consent forms were completed for each participant, one of which was given to the participant. Study staff read the informed consent form aloud and assessed potential participants’ comprehension of the study throughout the consent process by asking questions to gauge understanding of the study. Consents were then signed by the participant and RA if the participants still wanted to enroll. Illiterate participants used their fingerprint as their signature and had another person not involved in the study sign the consent as their witness. Parental consents and pediatric assents were obtained for participants aged 14–17 years of age, who were included so that we could evaluate their FP use. Participants received an equivalent of about USD$2 for completing the survey.

Surveys were conducted by RAs and entered directly into electronic tablets using Open Data Kit (ODK) Aggregate software (version 2.0). Data collected were uploaded from the tablet to a secure and password-protected UNC Project-Malawi ODK server and study ODK database daily in real-time by the RAs and cross-checked by our data managers for accuracy and completeness. Data from the ODK database were exported to Stata for data cleaning and analysis. The ODK tablets, ODK database, and Stata database only include de-identified data and can only be accessed by study staff members.

Prior to EHS data collection, two community sensitization strategies were employed. First, village leaders from the selected villages were invited for an information session on the purpose and procedure of EHS, with the plan that the leaders would then sensitize their villages. Travel costs to information sessions were reimbursed. Second, HSAs conducted sensitization in the villages prior to the team arriving in each village. These measures helped to ensure that the community was aware and welcoming of the RAs.

### 2.4. Study Period

Implementation of this study was originally planned to occur over 12 months (March 2020–February 2021). However, this time period was ultimately extended to 22 months because the study had to pause activities in April 2020 due to COVID-19 restrictions implemented by the Malawi MoH, after study implementation had started at six health facilities (Chileka, Kabudula, and Nkhoma in Lilongwe District, and Matawale, Namasalima, and St. Luke’s in Zomba District). Three of these facilities were assigned to Model 1 (St. Luke’s, Kabudula, and Namasalima), and the other three (Chileka, Matawale, and Nkhoma) to Model 2. During this COVID-19 pause, the six facilities were asked to stop community-based screening (if randomized to Model 2) and revert back to VIA screening at the facility; however, a few facilities still continued to offer HPV self-collection and thermal ablation when possible. We restarted implementation of the study in August 2020 when COVID-19 restricted were eased. We conducted refresher trainings in the study procedures at all 16 health facilities, first at the six facilities that had already started implementation in August 2020. By November 2020, all facilities were implementing study activities.

### 2.5. Study Randomization

The 16 selected facilities were each assigned a code and randomized 1:1 by our study biostatistician (MC) within each of the four strata (Table 1) using the codes [12]. This randomization resulted in four facilities from each district assigned to Model 1 and the other four facilities to Model 2. However, after randomization, we learned that neither facility in the first strata (Bwaila District Hospital and Zomba Central Hospital) could implement Model 2 because their HSAs were based at the hospital and did not have the capacity to perform community-based work. Therefore, they were dropped from the analysis for this manuscript, but kept in the study so that we could evaluate our implementation outcomes (acceptability, feasibility, etc.) at their sites for other study-related analyses. Of the 14 remaining facilities for this analysis, seven were assigned to Model 1 (three in Lilongwe, four in Zomba) and seven to Model 2 (four in Lilongwe, three in Zomba).

### 2.6. Power Calculations and Statistical Analyses

A study performed between 2011–2015 found that only 26.5% of Malawian women had ever undergone VIA for primary cervical cancer screening [14]. We estimated that with the introduction of clinic-based HPV self-collection in Model 1, at least 40% of eligible women in its catchment areas could be screened during study implementation. We then estimated that with the addition of community-based HPV self-collection to reach unscreened women, we could approach Malawi’s target rate of 80% of eligible women ever screened in Model 2 [15]. We collected data from potential facilities in Lilongwe and Zomba Districts and were able to estimate an average cluster size of 8000 women eligible for cervical cancer screening per health facility, with a coefficient of variation of the cluster sizes of 0.35. To estimate our intra-cluster correlation coefficient (ICC), we reviewed other similar studies from Malawi and found that their ICC ranged from 0.004–0.20 [12,16,17]. We therefore estimated that our ICC would range from 0.12 to 0.19.

We initially calculated ed that with 16 randomized facilities (8 per Model), we would have at least 80% power to detect a difference of at least 25–30% between our 2 Models, even if the proportion of women screened in Model 1 was as low as 40%. For example, if Model 1 screened 40% of eligible women, there would be 80% power to detect a difference if Model 2 screened at least 70% of women if the ICC was 0.18. If the ICC was 0.12, we would have 80% power to detect a 25% difference between Model 1 and Model 2 if Model 1 screened 40% of women.

However, after learning that Bwaila District Hospital and Zomba Central Hospital could not be assigned to Model 2, we dropped them from our sample size calculations, which decreased our average cluster size to 3400 eligible women. Assuming an ICC of 0.15, a coefficient of variation of cluster sizes of 0.35, and seven randomized facilities per Model, we would have at least 83% power to detect a difference of at least 30% between our 2 Models, even if the proportion of women screened in Model 1 was as low as 40%. All power calculations were calculated in R (Version 3.5.1, clusterPower package, Vienna, Austria).

The weighted proportions for the primary and secondary outcomes were based on the probability of selecting a village among villages in a facility catchment area using simple random sampling and selecting a household within a selected village using simple random sampling to account for the two-stage sampling method used in selecting the households. Our weights were therefore the inverse of the joint probability of selecting a village within a facility catchment area and selecting a household within a village.

The effect of the Model 1 compared to the Model 2 was assessed via logistic regression, adjusted for district, facility size, and facility location. The regression model accounted for health facility clustering by stratification, and the small number of clusters using the weights. Because of the small numbers for secondary outcome #1 (treatment-eligible women who received thermocoagulation), logistic regression was not performed for that comparison.

## 3. Results

**Figure 1:**
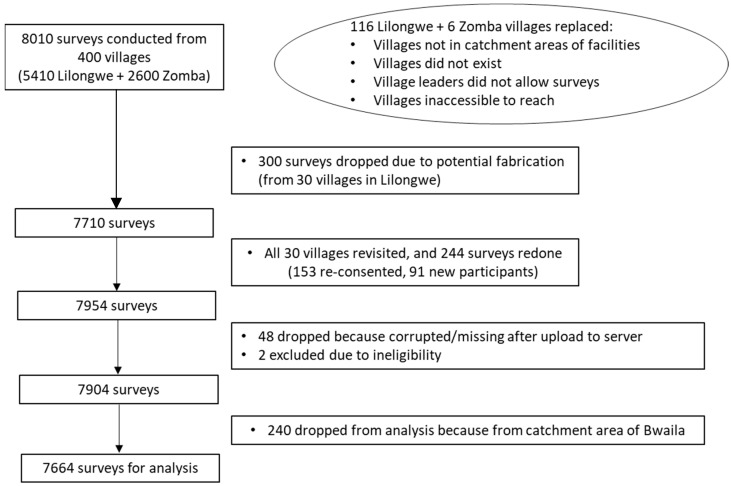
Schema showing how the final sample of 7664 surveys were derived for this analysis.

Of the 400 villages initially selected for interview, 116 villages in Lilongwe and six villages in Zomba had to be replaced once surveying was initiated (Figure 1). Twenty surveys were conducted in each village, with the exception of one village in Lilongwe, which had 30 surveys completed due to field error (Chinthankhwa village in Chiwamba), resulting in 8010 surveys in total. Towards the end of data collection, it was discovered that a RA in Lilongwe had not collected data as per study procedures. Therefore, the study team dropped all data collected from this RA, which included 300 surveys from 30 villages in Lilongwe district. Repeat sampling of these 30 villages was conducted, and we collected 244 replacement surveys, resulting in a total of 7954 surveys. Upon data cleaning, 48 surveys were noted to be corrupted or missing after upload, and two surveys were excluded due to ineligibility discovered after data collection (participants had hysterectomies prior to program start), resulting in 7904 surveys. Because Bwaila Hospital could not be randomized, the 240 surveys from its catchment area were not included in this analysis, resulting in a total of 7664 surveys for analysis.

### 3.1. Respondent Characteristics for the Endline Household Survey

Respondents of the EHS were nearly evenly split between the three age groups of 15–24 years, 25–34 years, and 35–50 years (Table 2). Most (91.8%) had attended some school and been pregnant (93.0%), and 34.1% had only a grass roof on their house, indicating low socioeconomic status. Very few (1.9%) had ever smoked tobacco, and 9.5% were known to be HIV-infected. The only sociodemographic differences between respondents from the catchment areas of Models 1 and 2 were that Model 1 participants were more likely to be single/never married (13.7% vs. 7.2%, *p* = 0.029) and were able to get to the nearest health facility in less than 1 h (41.7% vs. 26.5%, *p* = 0.049).

### 3.2. Endline Household Survey Responses for Cervical Cancer Screening (Primary Outcome) and Thermocoagulation Treatment (Secondary Outcome #1)

During our survey, only the 5138 respondents who were at least 25 years of age were asked questions about cervical cancer screening services, since Malawi MoH guidelines do not recommend such screening until that age (Table 3). We found that 33.1% of respondents in Model 1 had ever been screened for cervical cancer (Primary Outcome), compared to 42.5%, but this difference was not significant (*p* = 0.096).

A higher percentage of respondents in Model 1 (83.5%) remembered the approximate date when they were last screened for cervical cancer than Model 2 (67.4%, *p* = 0.046), which is notable because only those who recalled this date (n = 1185) were asked if they had undergone self-collection for cervical cancer since March 2020. Of the 1185 respondents, a lower proportion of 22.8% in Model 1 (n = 141) versus 50.5% of respondents in Model 2 (n = 471) said that they had underdone self-collection for cervical cancer (*p* = 0.023) and likely performed through our study as no other programs were known to be offering HPV self-collection in these facility catchment areas during the study implementation.

Of the 612 who underwent self-collection, 29.7% in Model 1 versus 21.6% in Model 2 said that they had undergone VIA (*p* = 0.284), and of the 118 who said that they had undergone VIA, 71.9% versus 57.1% (*p* = 0.517) said they also received treatment after VIA (Secondary Outcome #1) for Models 1 and 2, respectively.

The ICC for those participants who responded to the question about ever being screened for cervical cancer was 0.080 (95% CI 0.039–0.158) at the facility level. The responses about ever being screened for cervical cancer by facility catchment area are presented in Table A1.

### 3.3. Endline Household Survey Responses for Family Planning Use, Including Modern Family Planning Use (Secondary Outcome #2)

When asked about FP services, 6448 (83.1%) responded that they had ever used such services, which was not different between the 2 Models (Table 4). Of the 6448 who had ever used FP services, 60.6% (n = 3787) had used a modern FP method since our study was implemented in March 2020, but the difference between models was not significant (58.6% vs. 62.4%, *p* = 0.297). However, when asked which method they were currently using as their primary FP method (n = 3791; Secondary Outcome #2), Model 1 respondents were significantly less likely to report using a method that is considered a modern FP method (89.0%) than Model 2 respondents (96.5%, *p* = 0.034), which suggests that there was confusion about the question regarding modern FP-method use since March 2020.

Among those who said that they had used a modern FP method since March 2020, 34.4% (Model 1: 32.0% vs. Model 2: 36.5%, *p* = 0.287) responded that it was the first time that they had used this method, and 94.2% (Model 1: 94.1% vs. Model 2: 94.3%) said that they received the method they wanted to receive. Over half (56.2%) responded that they were advised to seek a cervical cancer screening service during a FP visit (Model 1: 55.7% vs. Model 2: 56.7%, *p* = 0.906).

The ICC for those participants who responded to the question about their primary FP method was 0.0032 (95% CI 0.0007–0.0136) at the facility level. The current use of modern FP method by facility catchment area is presented in Table A2.

### 3.4. Adjusted Results from the Endline Household Survey

For our adjusted analyses, we evaluated the effect of district, facility type (urban versus rural), and facility size (hospital versus health center) on the proportion of eligible women aged 25–50 years who had ever received CCS (Table 5) and who were currently using a modern FP method (Table 6).

Participants from Zomba district were more likely to have ever received CCS (0.507; 95% CI 0.445, 0.568; *p* < 0.001) than participants from Lilongwe District (0.311; 95% CI 0.273, 0.352), but there was no difference by closest facility type or size. Participants whose closest health facility was a hospital were more likely to be using a modern FP method (0.769; 95% CI 0.725, 0.808) than those whose closest health facility was only a health center (0.696; 95% CI 0.651, 0.738; *p* = 0.019), but there was no difference by district or facility type.

After adjusting for district, facility type, and facility size (Table 7), we found that women in Model 2 were more likely to have ever received CCS than in Model 1 (adjusted OR 1.73; 95% CI 1.29, 2.33). Women in Model 2 were also more likely to be using a modern FP method (adjusted OR 1.41; 95% CI 1.01, 1.98).

## 4. Discussion

In our study population, the integration of HPV-based CCS with FP services at both the clinic and community levels (Model 2) led to a significantly higher proportion of eligible women ever being screened for CCS and to be currently using a modern FP method, than when these services were only integrated at the clinic level (Model 1). A significantly higher proportion of women were screened for CCS through HPV self-collection in Model 2 than in Model 1 during the study implementation. We believe that the higher proportion of women who performed HPV self-collection in Model 2 is attributable to the community-based screening performed through our project since no other programs were implementing HPV self-collection in the catchment areas of the 14 facilities surveyed.

We were surprised that the proportion of women ever screened for CCS in the Model 1 facility catchment areas was only 33.1%, since a study between 2011–2015 found that 26.5% of Malawian women had been screened at least once in their lifetime by VIA [14]. We hypothesized that we could screen 40% of eligible women in Model 1 during the study, but that was a gross overestimate. However, we still found a significant difference between models for the proportion ever screened because the proportion was so low in Model 1, and our ICC was lower than expected.

The low proportion of women ever screened in Model 1 could be due to differences in how the Malawi MoH calculated their estimates for screening [18], a population which was more under-screened in our EHS than in their estimates, the failure to reach previously unscreened patients in Model 1, and/or misunderstanding of our survey question. In Model 2, we found that 42.5% of eligible EHS participants had ever been screened for CCS, suggesting that even if we failed to screen many new women in Model 1, we significantly increased the proportion of women ever screened in Model 2 by bringing HPV self-collection into the community.

One major related study limitation is that we did not perform a baseline household survey prior to project implementation due to timeline and financial constraints. We thought we could calculate the proportion of women screened through our study in our EHS by first asking participants if they had ever been screened for cervical cancer, then asking them if they had been screened via self-collection since our project started (in March 2020), and finally calculating the difference between those responses. However, only 66% of the EHS respondents ever-screened recalled the last date of their CCS, and only these respondents were asked about self-collection.

Therefore, we could not determine if those who did not recall their last screening date were screened through our program. In addition, some facilities reverted to VIA-based screening during the COVID-19 study pause, when they had no electricity and our solar UPS back-up systems failed (which occurred frequently, particularly at Chiwamba), and/or when we ran out of HPV self-collection kit supplies or cartridges (which we had difficulty procuring because of production and shipping delays). Those screened primarily with VIA through our study were not well-captured in our EHS, so we do not know the exact proportion of women screened by either VIA or HPV through our study and whether they received the treatment they needed if they screened positive. Finally, the data collected in the EHS was mostly by self-report and therefore subject to social desirability bias and recall bias, although the research assistants tried to verify responses in the health passport when possible.

The strengths of our study included: our cluster randomized design; implementation of our models in health facilities of different sizes, locations, and types; and strong partnership with the Malawi MoH and the district health management teams of the two districts, which enabled us to implement our project successfully, despite the many challenges that the COVID-19 pandemic brought upon our teams.

Prior studies have found that the integration of FP service provision with CCS leads to increased uptake of both services. A review published in 2017 identified six countries in SSA (Kenya, Nigeria, Tanzania, Uganda, Zambia, and Zimbabwe) that were integrating CCS with FP, although all were using VIA for primary screening and cryotherapy for treatment at that time [19]. The review highlighted that after CCSPT was introduced in Uganda, uptake for both intrauterine devices and implants increased three-fold and half of those who received any contraceptive were also screened for cervical cancer. A few other studies about FP-CCS integration have been published since, including articles from Guinea, Cameroon, and India [20,21,22], all of which found the integration to be feasible and acceptable, although data has been limited on its cost-effectiveness [21]. The study from India focused on sex workers and was the only one that used a community-based approach, although their approach differed from ours in that VIA was used for screening [22].

Another study from Malawi found that integrating FP and CCS into HIV care is acceptable and increased uptake of both FP and CCS among women living with HIV [23]. It also found that their integrated services required minimal additional resources over those needed for HIV care alone and that patient flow improved. Unlike our study, it used VIA as its primary screening method. Our team is currently performing budget and cost analyses to evaluate whether the additional resources needed to perform HPV-based screening are offset by the increased sensitivity and specificity for high grade cervical dysplasia when compared to VIA-based screening. Our team has already found that the HSAs in Model 2 spent 75% more time on FP services after CCS integration through our study when compared to pre-implementation, with no significant decrease on time devoted towards other activities [24]. Additional analyses are being performed to compare the acceptability, feasibility, and appropriateness of our two models, both for health-facility staff and women who underwent self-collection, and they will explore potential social behavior changes at the facility and community levels.

## 5. Conclusions

FP-CCS integration with HPV self-collection offered in both clinic and community-based settings was associated with a significantly higher proportion of eligible women ever being screened for cervical cancer in our adjusted analyses, as well as a higher proportion of women currently using a modern FP method, than when these services were only integrated at the clinic level. Now that the WHO has recommended HPV-based screening as the preferred cervical cancer screening method and that HPV self-collection should be offered as a screening option, policymakers, providers, and researchers around the world should consider evaluating whether the HPV-based FP-CCS implementation models utilized in our study could also be successfully implemented in their settings.

## Figures and Tables

**Table 1:** Selected sites with their randomized model number, within their health facility stratum.

Strata	Lilongwe District	Model	Zomba District	Model
Central or District Hospital	Bwaila District Hospital	1	Zomba Central Hospital	2
CHAM ^1^ Hospital	Nkhoma Hospital	2	St. Luke’s Hospital	1
Urban Health Center	Kawale Health Center	2	Matawale Health Center	2
	Area 18 Health Center	1	Zomba City Clinic	1
Rural Health Facility	Kabudula Rural Hospital	1	Domasi Rural Hospital	2
	Lumbadzi Health Center	2	Namasalima Health Center	1
	Chileka Health Center	2	Ngwelero Health Center	1
	Chiwamba Health Center	1	Likangala Health Center	2

^1^ Christian Health Association of Malawi.

**Table 2:** Characteristics of the Endline Household Survey respondents, total and by model.

			Model				
	Total (n = 7664)	Weighted %	Model 1: Clinic-Only (n = 3815)	Weighted %	Model 2: Clinic + Community (n = 3849)	Weighted %	Weighted *p*-Value
Age							
15–24 years	2526	32.1	1274	32.4	1252	31.9	
25–34 years	2655	34.8	1314	35.6	1341	34.0	0.615
35–50 years	2483	33.1	1227	32.0	1256	34.1	
Marital status							
Single/never married	484	10.4	236	13.7	248	7.2	
Married	6168	78.2	3071	75.8	3097	80.3	0.029 *
Divorced/Separated/Widowed	1012	11.4	508	10.4	504	12.4	
Ever attended school							
Yes	6786	91.8	3370	91.6	3416	92.0	0.810
No	878	8.2	445	8.4	433	8.0	
Primary material of roof							
Iron	2869	65.9	1251	66.7	1618	65.2	0.825
Grass	4795	34.1	2564	33.3	2231	34.8	
Average time to get to the nearest health facility (in hours)							
<1 h	1417	33.9	762	41.7	655	26.5	
1–2 h	4003	46.8	2136	44.8	1867	48.6	0.049 *
>2 h	2244	19.3	917	13.5	1327	24.9	
# Pregnancies							
Never pregnant	492	7.0	240	7.1	252	6.8	
1 pregnancy	1522	20.2	783	21.5	739	18.9	0.317
2–4 pregnancies	3706	48.4	1814	45.6	1892	51.0	
≥5 pregnancies	1944	24.4	978	25.7	966	23.2	
HIV Status							
HIV negative	6795	88.7	3425	86.8	3370	86.9	
HIV positive	544	7.1	223	9.6	321	9.5	0.992
Don’t know	325	4.2	167	3.5	158	3.6	
Ever smoked tobacco							
Yes	125	1.9	66	1.9	59	1.8	0.904
No	7539	98.1	3749	98.1	3790	98.2	

Abbreviations: HIV = human immunodeficiency virus. * *p* < 0.05.

**Table 3:** Responses to cervical cancer screening and treatment questions among respondents aged 25–50 years, total and by model.

			Model				
		Weighted	Model 1: Clinic-Only (n = 2541)	Weighted	Model 2: Clinic + Community (n = 2597)	Weighted	Weighted *p*-Value
	Total (n = 5138)	%		**%**		**%**	
Ever screened for cervical cancer							
Yes	1798	38	570	33.1	1228	42.5	0.096
No (End Survey)	3340	62	1971	66.9	1369	57.5	
Remember when last screened for cervical cancer (n = 1798)							
Yes	1185	74.2	385	83.5	800	67.4	0.046 *
No	613	25.8	185	16.5	428	32.6	
Since March 2020, got a self-test for CCS (n = 1185; only asked to those who recalled last screening date)							
Yes	612	37.4	141	22.8	471	50.5	0.023 *
No	573	62.6	244	77.2	328	49.5	
Underwent VIA after self-test? (n = 612)							
Yes	118	23.9	19	29.7	99	21.6	0.284
No	494	76.1	122	70.3	372	78.4	
Received treatment after VIA (n = 118)							
Yes	70	62.4	**8**	71.9	62	57.1	0.517
No	48	37.6	11	28.1	37	42.9	

Abbreviations: CCS = cervical cancer screening; VIA = visual inspection with acetic acid. * *p* < 0.05.

**Table 4:** Responses to family-planning questions among respondents aged 15–50 years, total and by model.

			Model				
	Total	Weighted	Model 1: Clinic-Only	Weighted	Model 2: Clinic + Community	Weighted	Weighted
	(n = 7664)	%	(n = 3815)	%	(n = 3849)	%	*p*-Value
Ever used any FP services							
Yes	6448	83.1	3204	82.3	3244	83.9	0.418
No	1216	16.9	611	17.7	605	16.1	
Since March 2020, ever used a modern FP method (n = 6448)							
Yes	3787	60.6	1854	58.6	1933	62.4	0.297
No	2661	39.4	1350	41.3	1311	37.6	
First time ever received this (modern) FP method (n = 3786)							
Yes	1405	34.4	659	32	746	36.5	0.287
No	2391	65.6	1204	68	1187	63.5	
Received desired FP method (n = 3786)							0.969
Yes	3569	94.2	1747	94.1	1822	94.3	
No	227	5.8	116	5.9	111	5.7	
Current primary FP method (n = 3791)							
Modern FP method **	3628	92.9	1778	89	1850	96.5	0.034 *
Not a modern FP method ***	163	7.1	85	11	78	3.5	
Advised to seek cervical cancer screening services during FP visit (n = 3796)							
Yes	2393	56.2	1093	55.7	1300	56.7	0.906
No	1403	43.8	770	44.3	633	43.3	

Abbreviations: FP = family planning. * *p* < 0.05. ** Response includes: female sterilization (n = 103), intrauterine device (n = 50), implant (n = 908), injectable (n = 2306), oral contraceptives (n = 212), emergency contraceptive pill (n = 4), condoms (n = 45). *** Response includes: withdrawal (n = 2), lactational amenorrhea method (n = 4), cycle beads (n = 1), other natural family planning methods (n = 68), don’t know (n = 78), and declined to respond (n = 8).

**Table 5:** Proportion of eligible women aged 25–50 years who have ever received cervical cancer screening by their district and the type and size of their closest health facility.

Strata	Total	Ever Received Cervical Cancer Screening	Weighted Proportion	*p*-Value
			(95% Confidence Interval)	
District				
Lilongwe	3368	1009	0.311 (0.273, 0.352)	<0.001 *
Zomba	1770	789	0.507 (0.445, 0.568)	
Facility Type				
Urban	784	356	0.396 (0.321, 0.476)	0.364
Rural	4345	1442	0.357 (0.324, 0.392)	
Facility Size				
Hospital	1735	715	0.408 (0.355, 0.464)	0.407
Health Center	3403	1083	0.375 (0.321, 0.432)	

* *p* < 0.05.

**Table 6:** Proportion of eligible women aged 15–50 years using a modern family planning method by their district and the type and size of their closest health facility.

Strata	Total	Using a Modern Family Planning Method	Weighted Proportion	*p*-Value
			(95% Confidence Interval)	
District				
Lilongwe	3248	2363	0.691 (0.636, 0.741)	0.167
Zomba	1751	1265	0.734 (0.698, 0.769)	
Facility Type				
Urban	726	505	0.683 (0.618, 0.742)	0.088
Rural	4273	3123	0.740 (0.712, 0.765)	
Facility Size				0.019 *
Hospital	1662	1242	0.769 (0.725, 0.808)	
Health Center	3337	2383	0.696 (0.651, 0.738)	

* *p* < 0.05.

**Table 7:** Adjusted Odds Ratios for comparing outcomes for Model 2 (Clinic + Community-based cervical cancer screening-family planning integration) versus Model 1 (Clinic-Only cervical cancer screening-family planning integration).

Outcome	Adjusted Odds Ratio (95% Confidence Interval)
Proportion Ever Received Cervical Cancer Screening	1.73 (1.29, 2.33)
Proportion Currently Using a Modern Family Planning Method	1.41 (1.01, 1.98)

## Data Availability

This study’s data will be housed at USAID’s Development Data Library at data.usaid.gov and made publicly available 1 year after all study analyses have been completed.

## Supplementary Materials

The following supporting information can be downloaded at: https://www.mdpi.com/article/10.3390/cancers15102797/s1, File S1: Self-Sample Collection Instructions.

## Appendix A

**Table A1:** Facility-specific weighted proportions for ever received cervical cancer screening.

Health Facility	Total	Ever Received Cervical Cancer Screening	Weighted Proportion (95% CI)	Model
Zomba City Clinic	142	82	0.599 (0.519, 0.675)	1
Nkhoma Mission Hospital	703	376	0.543 (0.479, 0.606)	2
Domasi Rural Hospital	293	155	0.536 (0.478, 0.593)	2
Chileka Health Center	90	43	0.525 (0.385, 0.661)	2
Matawale Health Center	247	131	0.517 (0.422, 0.610)	2
Likangala Health Center	452	196	0.453 (0.373, 0.536)	2
Lumbadzi Health Center	524	211	0.422 (0.342, 0.507)	2
St Luke’s Mission Hospital	113	42	0.407 (0.338, 0.479)	1
Namasalima Health Center	177	50	0.401 (0.278, 0.538)	1
Kawale Health Center	288	116	0.372 (0.309, 0.440)	2
Ngwelero Health Center	346	133	0.358 (0.293, 0.429)	1
Area 18 Health Center	107	27	0.251 (0.163, 0.366)	1
Kabudula Mission Hospital	626	142	0.224 (0.179, 0.277)	1
Chiwamba Health Center	1030	94	0.090 (0.073, 0.111)	1

**Table A2:** Facility-specific weighted proportions for modern family planning use.

Health Facility	Total	Using Modern Family Planning Method	Weighted Proportion (95% CI)	Model
Kabudula Rural Hospital	575	457	0.796 (0.751, 0.836)	1
Domasi Rural Hospital	291	215	0.791 (0.702, 0.858)	2
Lumbadzi Health Center	501	376	0.783 (0.710, 0.841)	2
Matawale Health Center	225	167	0.779 (0.670, 0.859)	2
Chileka Health Center	75	56	0.748 (0.682, 0.804)	2
Zomba City Clinic	107	68	0.728 (0.653, 0.792)	1
Nkhoma Mission Hospital	677	487	0.723 (0.663, 0.776)	2
Kawale Health Center	281	198	0.722 (0.646, 0.788)	2
Ngwelero Health Center	340	239	0.719 (0.650, 0.779)	1
Likangala Health Center	480	351	0.718 (0.633, 0.790)	2
Namasalima Health Center	189	139	0.706 (0.628, 0.774)	1
Chiwamba Health Center	1026	717	0.699 (0.648, 0.746)	1
St Luke’s Mission Hospital	119	86	0.690 (0.548, 0.803)	1
Area 18 Health Center	113	72	0.564 (0.478, 0.646)	1

## References

[1] Sung H., Ferlay J., Siegel R.L., Laversanne M., Soerjomataram I., Jemal A., Bray F. (2021). Global cancer statistics 2020: GLOBOCAN estimates of incidence and mortality worldwide for 36 cancers in 185 countries. CA Cancer J. Clin..

[2] UNAIDS Malawi 2021 Country Factsheet. https://www.unaids.org/en/regionscountries/countries/malawi.

[3] Walboomers J.M., Jacobs M.V., Manos M.M., Bosch F.X., Kummer J.A., Shah K., Snijders P.J.F., Peto J., Mejler L.M., Muñoz N. (1999). Human papillomavirus is a necessary cause of invasive cervical cancer worldwide. J. Pathol..

[4] Lei J., Ploner A., Elfström K.M., Wang J., Roth A., Fang F., Sundström K., Dillnetr J., Spalrén P. (2020). HPV Vaccination and the Risk of Invasive Cervical Cancer. N. Engl. J. Med..

[5] Bruni L., Saura-Lázaro A., Montoliu A., Brotons M., Alemany L., Diallo M.S., Afsar O.Z., LaMontagne D.S., Mosina L., Contreras M. (2021). HPV vaccination introduction worldwide and WHO and UNICEF estimates of national HPV immunization coverage 2010–2019. Prev. Med..

[6] World Health Organization (2021). WHO Guideline for Screening and Treatment of Cervical Pre-Cancer Lesions for Cervical Cancer Prevention.

[7] Einstein M.H., Smith K.M., Davis T.E., Schmeler K.M., Ferris D.G., Savage A.H., Gray J.E., Stoler M.H., Wright T.C., Ferenczy A. (2014). Clinical evaluation of the cartridge-based GeneXpert human papillomavirus assay in women referred for colposcopy. J. Clin. Microbiol..

[8] Serrano B., Ibáñez R., Robles C., Peremiquel-Trillas P., de Sanjosé S., Bruni L. (2022). Worldwide use of HPV self-collection for cervical cancer screening. Prev. Med..

[9] World Health Organization (2021). WHO Consolidated Guideline on Self-Care Interventions for Health, Version 2.1.

[10] Nishimura H., Yeh P.T., Oguntade H., Kennedy C.E., Narasimhan M. (2021). HPV self-collection for cervical cancer screening: A systematic review of values and preferences. BMJ Glob. Health.

[11] Arbyn M., Smith S.B., Temin S., Sultana F., Castle P. (2018). Detecting cervical precancer and reaching underscreened women by using HPV testing on self samples: Updated meta-analyses. BMJ.

[12] Tang J.H., Smith J.S., McGue S., Gadama L., Mwapasa V., Chipeta E., Chinkhumba J., Schouten E., Ngwira B., Barnabas R. (2021). Prevention of cervical cancer through two HPV-based screen-and-treat implementation models in Malawi: Protocol for a cluster randomized feasibility trial. Pilot Feasibility Stud..

[13] Advancing Partners & Communities (2014). Country Profile: Malawi Community Health Programs.

[14] Msayamboza K.P., Phiri T., Sichali W., Kwenda W., Kachale F. (2016). Cervical cancer screening uptake and challenges in Malawi from 2011 to 2015: Retrospective cohort study. BMC Public Health.

[15] Malawi Ministry of Health (2005). Malawi National Service Delivery Guidelines for Cervical Cancer Prevention.

[16] Mwapasa V., Joseph J., Tchereni T., Jousset A., Gunda A. (2017). Impact of mother-infant pair clinics and short-text messaging service (SMS) reminders on retention of HIV-Infected women and HIV-exposed infants in eMTCT care in Malawi: A cluster randomized trial. J. Acquir. Immune Defic. Syndr..

[17] Dovel K., Shaba F., Nyirenda M., Offorjebe O.A., Balakasi K., Phiri K., Hoffman R., Nichols B., Tseng C.-H., Bardon A. (2018). Evaluating the integration of HIV self-collection into low-resource health systems: Study protocol for a cluster-randomized control trial from EQUIP innovations. Trials.

[18] Malawi Ministry of Health (2016). National Cervical Cancer Prevention Program Strategy.

[19] White H.L., Meglioli A., Chowdhury R., Nuccio O. (2017). Integrating cervical cancer screening and preventive treatment with family planning and HIV-related services. Int. J. Gynaecol. Obstet..

[20] DeGregorio G., Manga S., Kiyang E., Manjuh F., Bradford L., Cholli P., Wamai R., Ogembo R., Sando Z., Liu Y. (2017). Implementing a fee-for-service cervical cancer screening and treatment program in Cameroon: Challenges and opportunities. Oncologist.

[21] Leno D.W.A., Diallo F.D., Delamou A., Komano F.D., Magassouba M., Niamy D., Tolno J., Keita N. (2018). Integration of family planning counselling to mass screening campaign for cervical cancer: Experience from Guinea. Obstet. Gynecol. Int..

[22] Reza-Paul S., Lazarus L., Maiya R., Venukumar K.T., Lakshmi B., Roy A. (2019). Delivering community-led integrated HIV and sexual and reproductive health services for sex workers: A mixed methods evaluation of the DIFFER study in Mysore, South India. PLoS ONE.

[23] Phiri S., Feldacker C., Chaweza T., Mlundira L., Tweya H., Speight C., Samala B., Kachale F., Haddad L. (2016). Integrating reproductive health services into HIV care: Strategies for successful implementation in a low-resource HIV clinc in Lilongwe, Malawi. J. Fam. Plann Reprod. Health Care.

[24] Chinkhumba J., Low D., Ziphondo E., Msowoya L., Rao D., Smith J.S., Schouten E., Mwapasa V., Gadama L., Barnabas R. (2022). Assessing community health workers’ time allocation for a cervical cancer screening and treatment intervention in Malawi: A time and motion study. BMC Health Serv. Res..

