# Supplementary material for: Results from Two HPV-Based Cervical Cancer Screening-Family Planning Integration Models in Malawi: A Cluster Randomized Trial
Source: Cancers (Basel). 2023 May 17;15(10):2797. doi: 10.3390/cancers15102797 (PMC10216717; doi:10.3390/cancers15102797)
Supplement: Supplementary file 1 [file cancers-15-02797-s001.zip › cancers-2354741-supplementary.pdf]

USAID FROM THE AMERICAN PEOPLE · UNIVERSITY OF MALAWI COLLEGE OF MEDICINE · msh Management Sciences for Health · THE UNIVERSITY of NORTH CAROLINA at CHAPEL HILL

Self-Sample Collection Instructions

Before you start, make sure you have:

- a brush with a blue handle
- a small tube labeled with your name

1

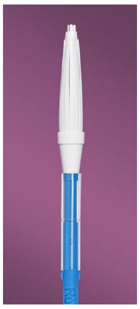

2

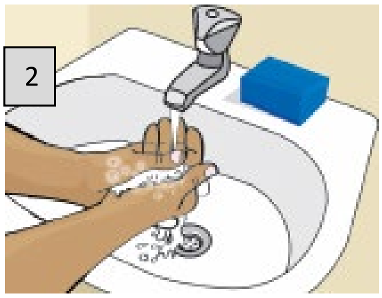

Find a private place to collect the sample.

Wash your hands with soap and water.

Open the envelope with the brush in it.

Remove the brush.

Try not to touch the white brush tip with your hands.

3

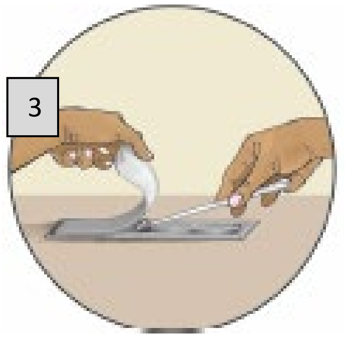

4

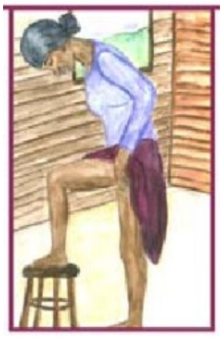

Stand, sit, or lie down in a comfortable position.

Some women find it helpful to squat with their legs apart.

Hold the lips of the vagina open with one hand.

Relax and gently push the brush into your vagina with the other hand until you feel a slight pressure.

5

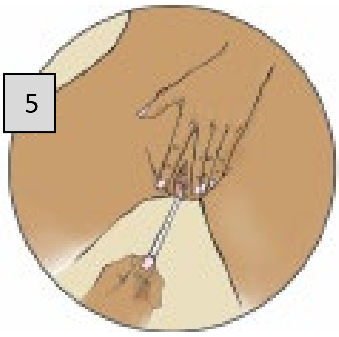

6

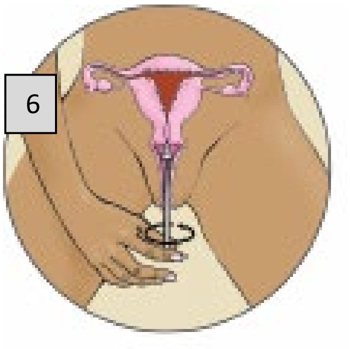

Gently turn the brush around 5 times while it is inside your vagina.

Slowly pull out the brush.

While holding the brush, remove the cap from the tube.

7

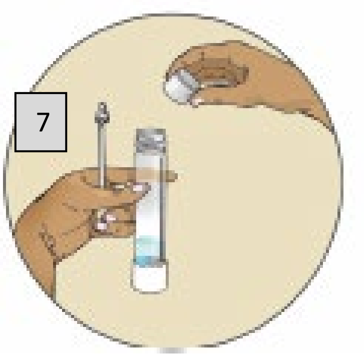

8

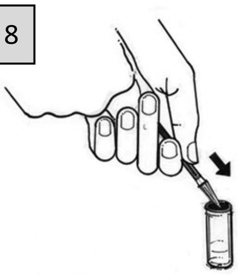

Notice that there is clear plastic on the handle near the white brush tip.
Push the clear plastic down so that the white brush tip falls into the tube.
Throw away the handle.

Put the cap back on the tube. Screw the lid on tightly. Wash your hands after collection. Then place the tube in the bio-hazard plastic bag provided.

9

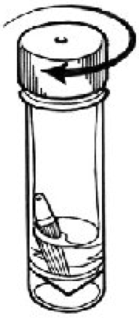

10

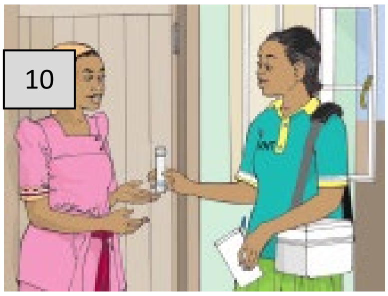

Return the tube to your provider.

You are finished!
